# Evaluation of ellagic acid and gallic acid as efflux pump inhibitors in strains of *Staphylococcus aureus*

**DOI:** 10.1242/bio.059434

**Published:** 2022-10-07

**Authors:** Nair Silva Macêdo, Cristina Rodrigues dos Santos Barbosa, Antonio Henrique Bezerra, Zildene de Sousa Silveira, Larissa da Silva, Henrique Douglas Melo Coutinho, Saeid Dashti, Bonglee Kim, Francisco Assis Bezerra da Cunha, Marcia Vanusa da Silva

**Affiliations:** ^1^ Laboratory of Semi-Arid Bioprospecting (LABSEMA), Department of Biological Chemistry - URCA, Crato, CE, 63105-000, Brazil; ^2^ Graduate Program in Biological Sciences, Federal University of Pernambuco - UFPE, Recife, PE, 50740-600, Brazil; ^3^ Laboratory of Microbiology and Molecular Biology (LMBM), Department of Biological Chemistry - URCA, Crato, CE, 63105-600, Brazil; ^4^ Department of Public Health, Ferdows School of Allied Medicine and Public Health, Birjand University of Medical Sciences, Birjand 9717853577, Iran; ^5^ Department of Pathology, College of Korean medicine, Kyung Hee University, Seoul 02447, Korea

**Keywords:** Phenolic acids, Tannins, Bacterial resistance, Multidrug resistance

## Abstract

The Gram-positive bacterium *Staphylococcus aureus* is responsible for a number of infections and has been described to exhibit resistance to antibacterial drugs through different resistance mechanisms. Among these, active efflux has been shown to be one of the main mechanisms of bacterial resistance associated with *S. aureus*. In this sense, the aim of the present study was to evaluate the ability of ellagic acid and gallic acid to reverse resistance by inhibiting the efflux pumps present in *S. aureus* strains IS-58 and K2068, which express the TetK and MepA flux pumps, respectively. In addition, the toxicity of both compounds was verified in *Drosophila melanogaster*. Broth microdilution assays were performed to obtain the minimum inhibitory concentration (MIC) values of ellagic acid and gallic acid, whereas efflux pump inhibition was tested using a subinhibitory concentration of standard efflux pump inhibitors, gallic acid and ellagic acid (MIC/8), where the ability of these compounds to decrease the MIC of ethidium bromide (EtBr) and antibiotics was verified. Toxicity was evaluated by mortality and negative geotaxis assays in *D. melanogaster*. Ellagic acid and gallic acid showed no direct antibacterial activity on *S. aureus* strains carrying the efflux pumps TetK and MepA. However, when we looked at the results for the TetK pump, we saw that when the two acids were associated with the antibiotic tetracycline, a potentiation of the antibacterial effect occurred; this behavior was also observed for the antibiotic ciprofloxacin in the MepA strain. For the efflux pump inhibition results, only the compound gallic acid showed potentiating effect on antibacterial activity when associated with the substrate EtBr for the IS-58 strain carrying the TetK efflux pump. Ellagic acid and gallic acid showed no toxicity on the model arthropod *D. melanogaster*. These results indicate the possible use of gallic acid as an adjuvant in antibiotic therapy against multidrug-resistant bacteria.

## Teaser

**Summary:** Gallic acid can be used as an adjuvant in antibiotic therapy against multidrug-resistant bacteria.

## INTRODUCTION

*Staphylococcus aureus* is an opportunistic pathogenic microorganism, characterized as a Gram-positive commensal bacterium in the form of cocci, being naturally present on mucosal surfaces. However, the occurrence of imbalance in its population can trigger severe acute and chronic infections, such as endocarditis, osteomyelitis, bacteremia, and skin and soft tissue disorders (Chang et al., 2020; Nair et al., 2014; Turner et al., 2019). Associated with these factors, *S. aureus* has developed resistance to the main classes of antibiotics existing in the clinic due to changes in its genetic elements.

This bacterial resistance has also intensified due to the high use of antibiotics, self-medication and treatment failure. In addition, antibacterial resistance is aggravated through natural biological factors, such as biochemical and genetic aspects, as there are several genes that are associated with bacterial resistance to antibiotics that determine the expression of genes encoding enzymatic resistance, target modification and active efflux (Kim et al., 2021; Pulingam et al., 2021). Active efflux is a resistance mechanism used by bacterial cells to extrude toxic substances, including antibiotics, out of the bacterial cell (Piddock, 2006). This mechanism is promoted by efflux pumps, which are transport proteins located in the plasma membrane and are energy-dependent, having places that allow the recognition of complementary molecules (Dey et al., 2022; Pulingam et al., 2021).

Among the efflux pumps present in *S. aureus*, we highlight the TetK efflux protein, belonging to the major facilitator superfamily (MFS), which is encoded in plasmids and confers resistance to antibiotics of the tetracycline class. (Chopra and Roberts, 2001). In contrast, the MepA efflux pump is part of the multidrug and toxic compound extrusion (MATE) family, being responsible for extruding several biocides such as benzalkonium chloride and ethidium bromide (EtBr), as well as antibiotics of the fluoroquinolone class such as ciprofloxacin, norfloxacin and moxifloxacin (Kaatz et al., 2005; Schindler et al., 2015; 2013).

Therefore, the investigation for new substances that act as efflux pump inhibitors (EPIs) has grown and received attention in pharmacological research, highlighting those that include the modification of bacterial resistance. Among these substances, the class of phenolic compounds is well investigated for their antimicrobial properties, owing to their large-scale presence in plants and their organs, such as flowers, leaves, seeds and fruits (Ozcan et al., 2014). Phenolic acids are part of a subgroup of phenolic compounds and are characterized by having a chemical structure consisting of a phenolic ring and a carboxylic acid organic function (Kahkeshani et al., 2019). These compounds have been described as viable alternatives in reversing bacterial resistance mechanisms, such as efflux pumps (dos Santos et al., 2021; Pinheiro et al., 2021).

Among them, ellagic acid and gallic acid are distributed in abundance in the vegetable kingdom, they are characterized as colorless or yellowish substances that have application in the pharmaceutical and food industry sectors (Amakura et al., 2000; Kahkeshani et al., 2019). Previous studies have already reported various pharmacological effects for these compounds, which include direct antibacterial activity, modification of antibiotic activity and reversal of the resistance phenotype associated with the MrsA, NorA, TetR and TetM efflux pumps (dos Santos et al., 2018; Jenic et al., 2021; Lima et al., 2016; Sivakumar et al., 2020). However, the effects of these compounds on reversing resistance by inhibiting the TetK and MepA efflux pumps have not yet been evaluated.

Determining the toxicity of substances that are candidates for potential EPIs is crucial to ensure the safety of these products. A model widely used to verify the toxicity and genotoxicity of substances is the arthropod *Drosophila melanogaster*, popularly known as the fruit fly, with advantages for carrying out these studies such as short life cycle, low maintenance cost and similarity with humans in relation to their physiological and neurological characteristics (Calap-Quintana et al., 2017; Reiter et al., 2001).

Therefore, this study aimed to evaluate the ability of the compounds ellagic acid and gallic acid to reverse the resistance of efflux pumps present in the *S. aureus* strains IS-58 and K2068, which express the TetK and MepA efflux pumps, respectively. In addition, it verified the toxicity of both compounds in *D. melanogaster*.

## RESULTS

### Antibacterial activity and modification of the MIC of antibiotics

When evaluating the antibacterial activity of gallic and ellagic acids through the minimum inhibitory concentration (MIC) test on the IS-58 and K2068 strains of *S. aureus*, it was observed that for both strains, the two compounds presented a MIC≥1024 µg/ml, which did not demonstrate direct antibacterial activity. This data is shown in Table 1.

**Table 1.**
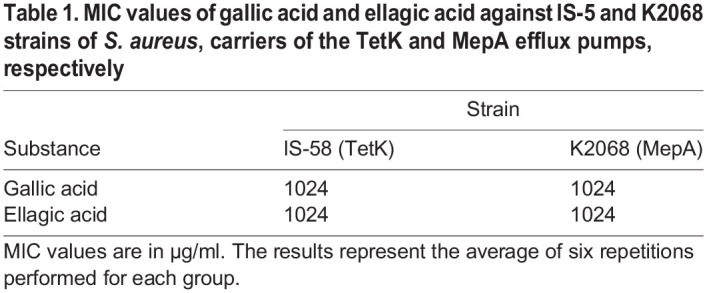
MIC values of gallic acid and ellagic acid against IS-5 and K2068 strains of *S. aureus*, carriers of the TetK and MepA efflux pumps, respectively

**Fig. 1.**
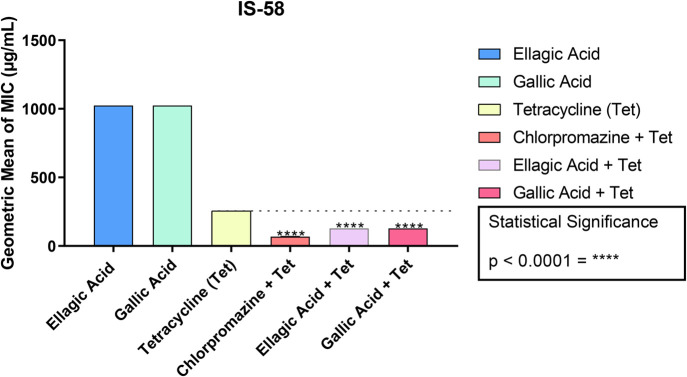
**Ability of ellagic and gallic acids to modify the MIC of the antibiotic tetracycline and the standard inhibitor chlorpromazine against the IS-58 strain of *S. aureus*.** These data represent the MIC values of the antibiotic alone (tetracycline) and the values corresponding to the association of the antibiotic with the standard inhibitor (chlorpromazine) and the natural compounds (gallic acid and ellagic acid) for the IS-58 strain of *S. aureus.* ****P<0.0001. Values are represented by the geometric mean±s.e.m. One-way ANOVA was used with Bonferroni post hoc test.

The assays to verify the modification of the MIC of antibiotics by the use of ellagic acid and gallic acid showed a reduction in the MIC of tetracycline from 256 to 128 µg/ml for both acids against the IS-58 strain of *S. aureus*, as shown in Fig. 1 and Table 2.

**Table 2.**
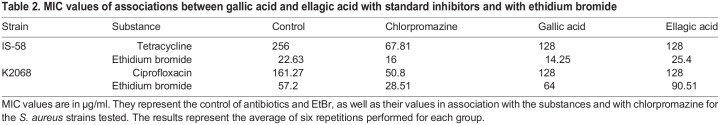
MIC values of associations between gallic acid and ellagic acid with standard inhibitors and with ethidium bromide

**Fig. 2.**
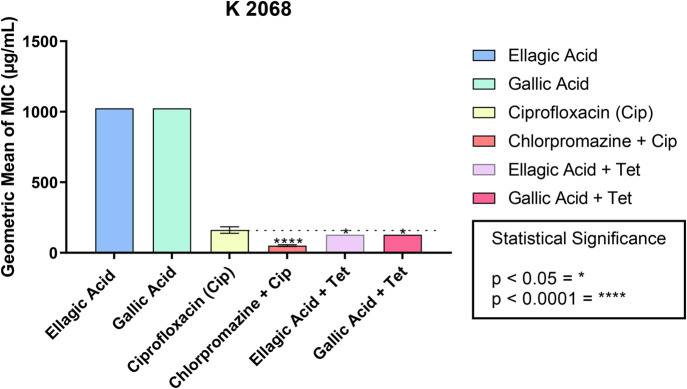
**Ability of ellagic and gallic acids to modify the MIC of the antibiotic ciprofloxacin and standard inhibitor chlorpromazine against the K2068 strain of *S. aureus*.** These data represent the MIC values of the antibiotic alone (ciprofloxacin, CIP) and the values corresponding to the association of the antibiotic with the standard inhibitor (chlorpromazine) and the natural compounds (gallic acid and ellagic acid) for the K2068 strain of *S. aureus*. *P<0.05; ****P<0.0001. Values are represented by the geometric mean±s.e.m. One-way ANOVA was used with Bonferroni post hoc test.

When evaluating the ability to modify the MIC value in association with ciprofloxacin, a potentiating effect of antibacterial activity was observed between phenolic acids and the antibiotic, characterized by a reduction in MIC from 161.27 to 128 µg/ml when associated with ellagic and gallic acids on the K2068 strain of *S. aureus* (Table 2, Fig. 2).

### Modification of MIC of EtBr in association with ellagic and gallic acids against IS-58 and K2068 strains of *S. aureus*

**Fig. 3.**
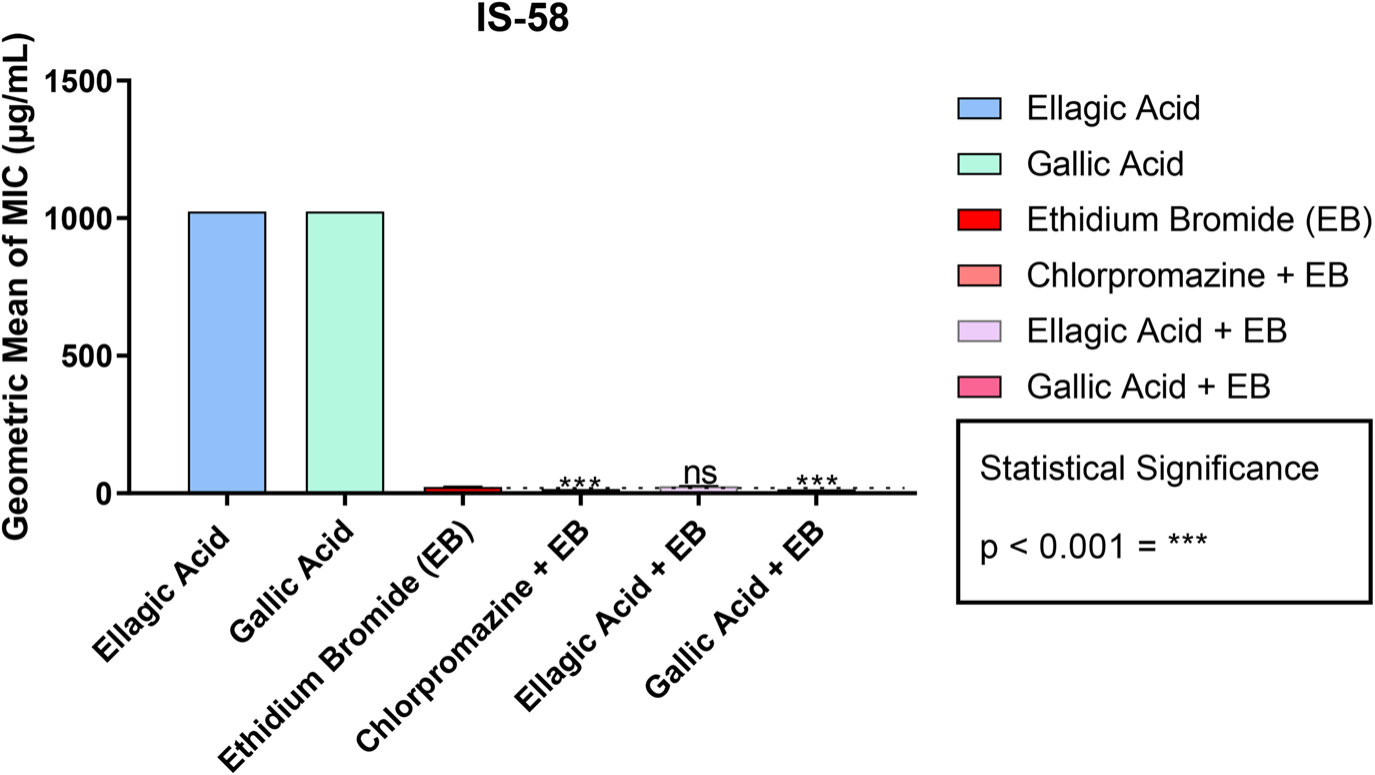
**Ability of ellagic and gallic acids to modify the MIC of ethidium bromide against the IS-58 strain of *S. aureus.*** These data represent the MIC values of the efflux pump substrate (ethidium bromide) and the values corresponding to the association of the substrate with the standard inhibitor (chlorpromazine) and the natural compounds (gallic acid and ellagic acid) for the IS-58 strain of *S. aureus.* ns, not significant; ***P<0.001. Values are represented by the geometric mean±s.e.m. One-way ANOVA was used with Bonferroni post hoc test.

The result of the association between EtBr and ellagic acid demonstrated an antagonism between the two substances, raising the MIC of EtBr from 22.63 to 25.4 µg/ml (Table 2, Fig. 3). This shows that ellagic acid does not have an inhibitory activity on the TetK efflux pump against the IS-58 strain. On the other hand, the association between EtBr and gallic acid showed a potentiating effect of the antibacterial activity between the substances, observed by the reduction of the MIC of EtBr from 22.63 to 14.25 µg/ml (Table 2, Fig. 3). This reduction suggests that gallic acid has an inhibitory effect on the TetK efflux pump mechanism present in the IS-58 strain of *S. aureus*.

**Fig. 4.**
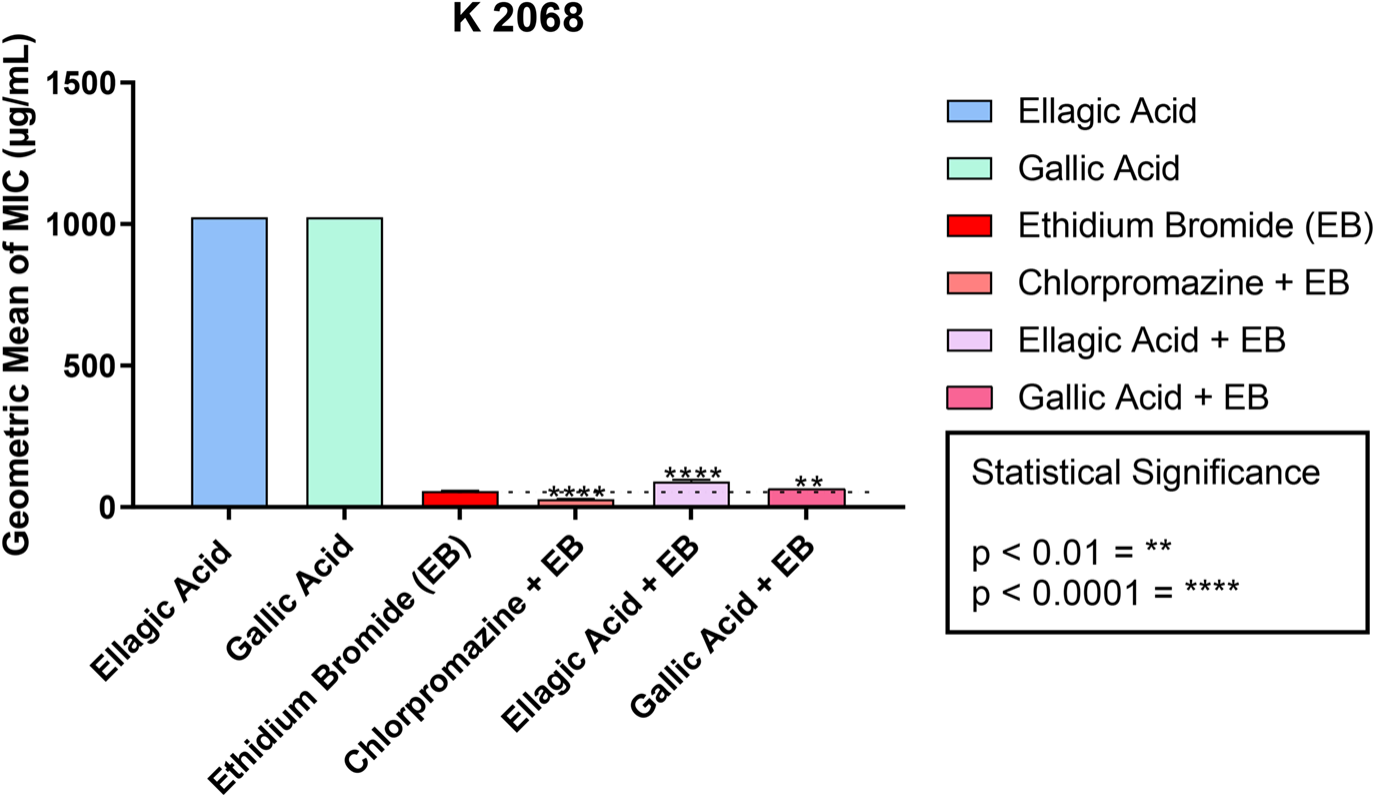
**Ability of ellagic and gallic acids to modify the MIC of ethidium bromide against K2068 strain of *S. aureus*.** These data represent the MIC values of the efflux pump substrate (ethidium bromide) and the values corresponding to the association of the substrate with the standard inhibitor (chlorpromazine) and the natural compounds (gallic acid and ellagic acid) for the K2068 strain of *S. aureus*. **P<0.01; ****P<0.0001. Values are represented by the geometric mean±s.e.m. One-way ANOVA was used with Bonferroni post hoc test.

In the modulation assays of EtBr MIC on the K2068 strain of *S. aureus*, the results of the association with ellagic acid and gallic acid showed an increase in the EtBr MIC value from 57.02 to 90.51 µg/ml and 64 µg/ml, respectively (Table 2, Fig. 4). These assays showed an antagonism between EtBr and the two acids, which suggests that these two substances do not present inhibitory activity of the MepA efflux pump mechanism that is present in the K2068 strain of *S. aureus*.

### Toxicity and negative geotaxis assays with *D. melanogaster*

**Fig. 5.**
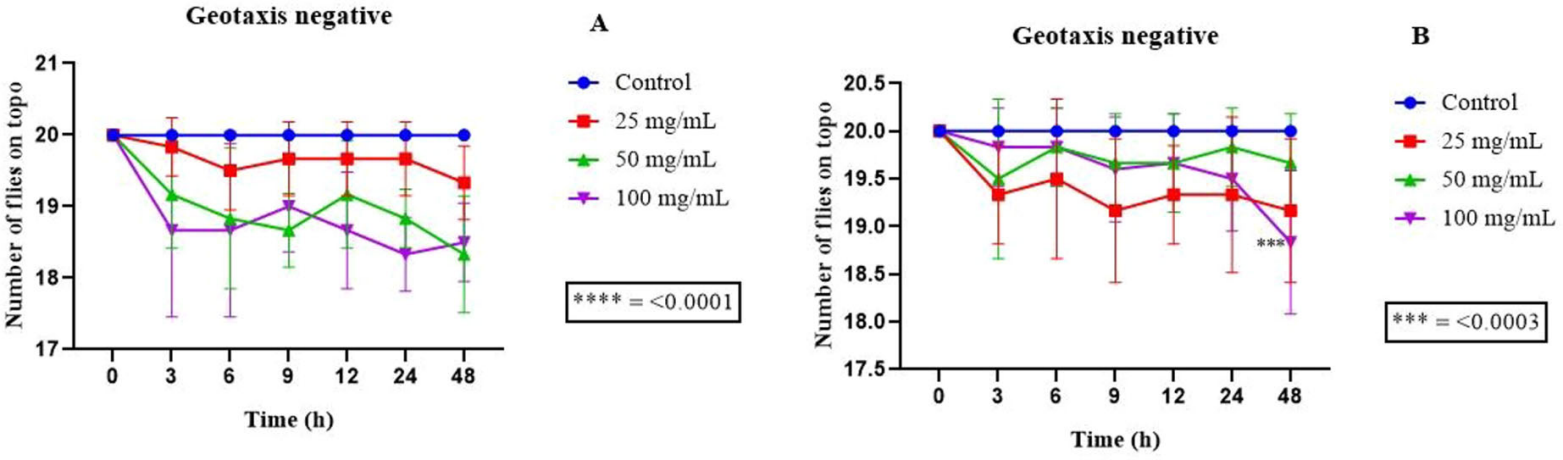
**Negative geotaxis assay using the *D. melanogaster* model.** Data represent results for negative geotaxis assays in which (A) ellagic acid and (B) gallic acid were used. The concentrations used were 25, 50 and 100 mg/ml. ***P<0.0003, and represents significance in relation to the control. Two-way ANOVA was used.

Toxicological tests showed that ellagic acid and gallic acid did not show toxicity against the model arthropod *D. melanogaster* at all tested concentrations of 25, 50 and 100 mg/ml, and did not affect the survival rates of flies over time of exposure to the isolates. Therefore, it was not possible to calculate the half maximal effective concentration (EC_50_). The damage to the locomotor capacity of *D. melanogaster* was evaluated using the negative geotaxis assay. According to the data obtained, no significant damage was observed in the locomotor capacity of *D. melanogaster* exposed to ellagic acid at any of the concentrations tested (Fig. 5). The data referring to the effect of gallic acid indicated that only the concentration of 100 mg/ml differed statistically from the control after 48 h of exposure, showing negative effects on the locomotor apparatus of the flies (Fig. 5).

## DISCUSSION

Ellagic acid and gallic acid did not show direct antibacterial activity on *S. aureus* strains carrying TetK and MepA efflux pumps. However, when we look at the results for the TetK pump, it appears that when the two acids were associated with the antibiotic tetracycline, there was a potentiating effect of the antibacterial activity, i.e. a lower concentration of the antibiotic was required to inhibit bacterial growth.

Previous trials demonstrated that ellagic acid reduced the MIC of tetracycline for an *Escherichia coli* strain that was an efflux pump carrier (Jenic et al., 2021). For gallic acid, there are results in the literature with the same strain used in this study, IS-58, which expresses the TetK efflux pump encoded by plasmid pT181. However, dos Santos et al. (2018) found that gallic acid did not differ statistically from the control when associated with the antibiotic tetracycline.

The results of the modulation of the antibiotic ciprofloxacin by ellagic acid and gallic acid to the MepA pump demonstrated an enhancement of the antibacterial activity. The MepA efflux protein is encoded through the bacterial chromosome, and its expression is regulated by MepR, characterized as a substrate-binding regulatory protein of the MarR family, in addition to acting as a MepA repressor (Kaatz et al., 2006; 2005). The K2068 strain of *S. aureus* expresses the MepA efflux pump, presenting a mutation in the operon region, where the MepA gene is located, but it does not have mutations in the topoisomerase associated with its resistance spectrum (Freitas et al., 2021b). The literature is still limited in terms of the functioning characteristics of MepA due to limitations associated with the identification of its substrates.

For the efflux pump inhibition results, only the compound gallic acid showed potentiating effect of antibacterial activity when associated with the substrate EtBr for the IS-58 strain carrying the TetK efflux pump, and its effect was similar to the effect of the standard inhibitor used, chlorpromazine. In the literature, chlorpromazine is used as a specific efflux pump inhibitor and acts as a competitive inhibitor, having its action place located close to the substrate of the antibiotic (Tintino et al., 2016). Gallic acid reduced the MIC of EtBr for *S. aureus* strains carrying the TetK, NorA and MrsA efflux pumps; these results may be associated with lower drug affinity and non-chelation with EtBr by gallic acid (dos Santos et al., 2018).

According to the evidence available in the literature, the accumulation and efflux of EtBr are good indicators of the involvement of efflux pumps in the resistance mechanism of *S. aureus* (Kalia et al., 2012). In our data, the EtBr assay confirms a possible effect of gallic acid in inhibiting the efflux pump activity present in the IS-58 strain, because in the presence of gallic acid, the MIC of EtBr was reduced, reflecting a strong interference in the efflux mechanism of EtBr by the compound.

The mechanisms of action by which phenolic acids operate to inhibit bacterial growth are associated with their structure-activity, with some factors being the basic chemical structure of the acid, the location and number of hydroxyl groups, as well as their substitutes in the phenolic ring, in addition to the esterification of the carboxyl group (Kahkeshani et al., 2019).

The interest in investigating the antibacterial effect of compounds isolated from natural products has grown significantly. However, studies on the adverse effects of these compounds on animals and humans are still limited, and it is imperative to study the safety and bioactivities of these substances to provide sufficient scientific data for their therapeutic application (Pinho et al., 2014).

In this study, the model organism *D. melanogaster* was used to investigate the effects of ellagic acid and gallic acid concerning the survival and locomotor ability of flies. This organism has been increasingly recognized as a model in response to dietary factors and assessment of impacts on its survival, life expectancy and locomotor performance (Piegholdt et al., 2016; Wagner et al., 2015).

The data from ellagic acid toxicity assays showed no toxic effects of this substance on *D. melanogaster*, which was reflected in both mortality and negative geotaxis assays. These results are in agreement with previous reports, which demonstrated that ellagic acid had no toxic effects on this organism, being responsible for prolonging the longevity of flies, reducing fecundity and increasing resistance to thermal shock, cold, hunger stress and oxidative stress (Kharat et al., 2020). Furthermore, the study by Khatri and Juvekar, (2016) demonstrated that ellagic acid improved locomotor performance and reduced toxicity on *D. melanogaster* subjected to a Parkinson's disease model. The gallic acid, on the other hand, did not show significant results on the survival of *D. melanogaster*. However, the effects on locomotor capacity were only observed at the highest concentration evaluated after 48 h of exposure. Previous studies have described neuroprotective effects of gallic acid in different models of neurodegeneration, neurotoxicity and oxidative stress in a model of Alzheimer's disease and Parkinson's disease using *D. melanogaster* (Jimenez-Del-Rio et al., 2010; Ogunsuyi et al., 2019). This phenolic acid also demonstrated a protective effect on *D. melanogaster* exposed to the organophosphate chlorpyrifos, which inhibits acetylcholinesterase, the main mechanism of action of this insecticide (Gomes et al., 2020). The results obtained by Nagpal and Abraham (2017) suggest that gallic acid has antioxidant and antimutagenic properties in *D. melanogaster* exposed to urethane-induced genotoxicity and oxidative stress.

In conclusion, ellagic acid and gallic acid did not show direct antibacterial activity on *S. aureus* strains carrying TetK and MepA efflux pumps. However, when the compounds were associated with antibiotics, a potentiation of the antibacterial activity was observed. Additionally, only gallic acid moderately decreased the MIC of EtBr on the TetK efflux pump. Ellagic acid and gallic acid showed no toxicity on the model arthropod *D. melanogaster*. However, molecular assays are necessary to understand the mechanism of action of substances on the studied strains.

## MATERIALS AND METHODS

### Microbiological tests

#### Bacterial strains

The *Staphylococcus aureus* strains used were IS-58 and K2068, which express the TetK and MepA efflux pumps, respectively. The strains were provided by Prof. Simon Gibbons (University of London) and were maintained in blood agar (Difco Laboratories, Brazil). Before the experiments, they were cultivated for 24 h at 37°C in a solid Brain Heart Infusion (BHI) agar (Acumedia Manufacturers, USA).

#### Culture medium

To realize the microbiological tests, the following culture media were used: BHI agar (Acumedia Manufacturers, USA), prepared according to the manufacturer’s instructions, and BHI (Acumedia Manufacturers, USA) prepared at the same concentration of 10%.

#### Chemicals and reagents

The antibiotics (tetracycline and ciprofloxacin), EtBr, ellagic acid and gallic acid were obtained from Sigma-Aldrich (St. Louis, MO, USA), and chlorpromazine (CPMZ) was obtained from Aché Pharmaceuticals Laboratories (Pernambuco, Brazil). The antibiotics norfloxacin and ciprofloxacin, as well as ellagic acid and gallic acid, were diluted in dimethylsulfoxide (DMSO) and in sterile water. The proportion of DMSO used was less than 5%. CPMZ and EtBr were dissolved in sterile distilled water. All substances were diluted to a standard concentration of 1024 µg/ml.

#### Determination of MIC

MIC was determined for ellagic acid and gallic acid according to the broth microdilution method proposed by Javadpour et al. (1996) with adaptations. The strains used in the tests were sown 24 h before the experiments. After this period, the bacterial inoculum was suspended in saline solution, corresponding to 0.5 on the McFarland scale, or approximately 1.5×10^8^ colony-forming units (CFU)/ml. Microtubes (Eppendorfs, Ala Artefacts Laboratories, Brazil) were filled with 900 µl of BHI and 100 µl of the inoculum and the plates were filled with 100 µl of the final solution. Microdilution was performed with 100 µl in serial dilutions up to the penultimate pit of the plate (1:1), being the last used as a growth control. The concentrations of the compounds ranged from 512 µg/ml to 8 µg/ml. After 24 h of incubation, readings were realized by adding 20 µl of resazurin (7-hydroxy-10-oxidophenoxazin-10-ium-3-one) (Exodo, Brazil). Resazurin was oxidized in the presence of the acid medium caused by bacterial growth, promoting the color change from blue to pink (Elshikh et al., 2016). The MIC was defined as the lowest concentration in which no growth could be observed (Andrews, 2001). The tests were performed in triplicate.

#### Evaluation of efflux pump inhibition by modification of MIC of antibiotics and EtBr

To observe whether ellagic acid and gallic acid acted as potential inhibitors of the TetK and MepA efflux pumps, a comparative study between the effects of the standard inhibitors of the efflux pumps was used, evaluating the ability of both compounds to decrease the MIC of EtBr and the antibiotics tetracycline and ciprofloxacin. The standard inhibitor chlorpromazine was used to provide the expression of the TetK and MepA pumps by the strains tested. The inhibition of the efflux pumps was tested using a subinhibitory concentration (MIC/8) of inhibitors and ellagic acid and gallic acid. In the tests, 170 µl of each bacterial inoculum suspended in saline solution, corresponding to 0.5 on the McFarland scale, or approximately 1.5×10^8^ CFU/ml, was added together with the inhibitors and ellagic acid and gallic acid (MIC/8) and completed with BHI. These were transferred to 96-well microdilution plates, to which 100 µl of antibiotic or EtBr were added in serial dilutions (1:1) ranging from 512 to 0.5 µg/ml. The plates were incubated at 37°C for 24 h and bacterial growth was evaluated with resazurin. The MIC of the controls was evaluated using only plates with tetracycline and with EtBr, and the tests were performed in triplicate.

#### Negative control

For the negative control, the last rank of microdilution plate cavities was used, in which only the culture medium and bacterial inoculum were added without the addition of the tested substances, to ensure the maximum formation of the number of bacterial colonies and to prove that the inoculum was properly added to the microtubes. However, these numbers were not included in the results due to the difficulty of measuring the number of colonies formed in a period of 24 h after the microbiological tests. The methodology used does not allow the counting of bacterial colonies; however, we used resazurin as a colorant to identify bacterial growth through oxidation-reduction.

### Toxicity tests

#### Drosophila melanogaster stock

*Drosophila melanogaster* (Harwich strain) was obtained from the National Species Stock Center, Bowling Green, OH, USA. Flies were raised in 340 ml glass jars (15 cm high and 6.5 cm in diameter) grown with the medium containing 83% corn mass, 4% sugar, 4% lyophilized milk, 4% soybean meal, 4% wheat or oat bran and 1% salt. When the mixture was cooked, 1 g of Nipagin (methylparaben) was added, which is used as an antibacterial to preserve the mixture for a longer period, having action on bacteria, fungi and yeast. After cooling in the growth flasks, 1 ml of solution containing *Saccharomyces cerevisiae* was added to keep the food moist to facilitate feeding the flies. The flies were raised in biochemical oxygen demand (BOD) photoperiod greenhouses at a temperature of 25±1°C and 60% relative humidity.

#### Survival tests

The ingestion bioassay method (Da Cunha et al., 2015) was used to assess the toxicity of ellagic acid and gallic acid. Adult flies (male and female aged approximately 4 days) in numbers of 20 were placed in 130 ml flasks, previously prepared with 1 ml of sucrose solution in distilled water, at a concentration of 20%, allowing the flies to be fed *ad libitum*. The other treatments received volumes of 25, 50 and 100 mg/ml of ellagic acid and gallic acid, and these volumes were diluted in a 20% sucrose solution. All bioassays were conducted in a BOD type greenhouse with a light and dark cycle every 12 h, temperature of 26±1°C and 60% relative humidity. The tests were performed in triplicate and the readings of mortality rates were taken at 3,6, 9, 12, 24 and 48 h (Da Cunha et al., 2015).

#### Negative geotaxy test

The damage to the locomotor system was determined by the negative geotaxy test, as described by Coulom and Birman (2004). Briefly, shortly after counting the mortality of the flies every 3, 6, 9, 12, 24 and 48 h, the negative geotaxy test was carried out concomitantly with the surviving flies, which consists of counting the number of flies that ascend in the column of the experiment's own glass, above 5 cm, in a time interval of 5 s. Assays were repeated twice at 1 min intervals. Results were presented as the mean time(s)±s.e.m. obtained in two independent experiments.

#### Statistical analysis

The central data and standard deviations of microbiological assays were obtained according to the methodology of Freitas et al. (2021a), on microbiological analysis in microdilution plates. The data were analyzed using the statistical program GraphPad Prism 6.01 through a one-way ANOVA test with a post hoc Bonferroni test (where *P*<0.05 was considered significant and *P*>0.05 non-significant). To analyze the toxicity data, a one-way ANOVA test was performed, followed by a Tukey’s multiple comparisons test. There was no statistical difference with the same concentration versus time.
